# The Breakfast Club: Enhancing Emergency Medicine Education Through Spaced Retrieval and Elaborative Interrogation Techniques

**DOI:** 10.1002/aet2.70168

**Published:** 2026-04-14

**Authors:** Abigail Alorda, Joseph R. Ray, Stephanie Cohen, Shayne Gue

**Affiliations:** ^1^ Department of Emergency Medicine HCA Healthcare/USF Morsani College of Medicine GME: HCA Florida Oak Hill Hospital Brooksville Florida USA; ^2^ EM Residency Program UCF/HCA Florida Healthcare GME (Greater Orlando/Osceola) Florida USA; ^3^ Emergency Medicine University of Central Florida College of Medicine Orlando Florida USA; ^4^ EM Residency Program BayCare Health System/St. Joseph's Hospital Tampa Florida USA; ^5^ Medical Education University of Central Florida College of Medicine Orlando Florida USA

## Abstract

**Background:**

Emergency medicine (EM) residencies present a challenging educational environment. Despite extensive research supporting cognitive learning strategies such as retrieval practice, spaced repetition, and elaborative interrogation in improving knowledge retention, these strategies remain underutilized in graduate medical education (GME) settings. There is a critical need to develop and implement novel teaching methodologies that enhance resident learning and retention without detracting from patient care. This study aims to address this need by integrating said techniques in an innovative curriculum titled “The Breakfast Club.” Prior research in non‐GME education settings has demonstrated the efficacy of these techniques in promoting deeper understanding and longer retention of complex topics. We hypothesize that incorporating these strategies will positively impact EM residents' comprehension and retention.

**Methods:**

The curriculum was implemented in a single‐institution EM residency program as a pilot study. Residents in the intervention group participated voluntarily in a hour‐long virtual session facilitated by one instructor utilizing cognitive learning techniques; residents in the control group participated only in the standard program curriculum. A pretest and postintervention assessment were used to compare knowledge comprehension and retention at short‐term and long‐term intervals. Questions were developed and reviewed by EM educators for content validity and quality assurance.

**Results:**

Our study revealed a trend toward greater knowledge translation. Residents who participated in a single Breakfast Club session (*n* = 7) demonstrated improved performance compared with baseline, scoring 77% on short‐term multiple‐choice question (MCQ) assessments and 69% on long‐term MCQ assessment, compared with a baseline score of 53%.

**Conclusion:**

A brief, small group intervention using cognitive learning techniques was easy to implement, required minimal resources, and engaged residents to improve their medical knowledge in gastrointestinal pathophysiology. Further exploration with a larger cohort and varying topics will help establish whether these techniques can serve as a cornerstone for evidence‐based teaching strategies in EM education.

**Abbreviations**
EM: emergency medicine
GME: graduate medical education
PGY: postgraduate year
ITE: in‐training exam
MCQ: multiple‐choice question

## 1 Need for Innovation

Graduate medical education (GME) aims to cultivate self‐directed lifelong learners who provide effective patient care [1]. Emergency medicine (EM) residency programs have a particular challenge in that they must prepare residents to evaluate and manage a wide spectrum of diseases and pathology. The In‐Training Exam (ITE) is one educational tool that is utilized to assess residents' likelihood of passing the American Board of Emergency Medicine (ABEM) Qualifying Exam [2]. Residency programs commonly utilize question banks and mandatory didactic sessions to prepare their learners for these high‐stakes exams, but residents report they still are not adequately prepared for the ABEM Qualifying Exam with these methods alone [1]. Additionally, board exam preparation constitutes only one of many time‐consuming requirements that residents must balance during training. High‐acuity clinical demands can also limit traditional didactic learning opportunities. One study reported that even after the implementation of the ACGME duty hours requirements, residents spent approximately 11.6% of their time on educational activities [3]. Therefore, it is crucial to maximize the effectiveness of that time.

## 2 Background

Despite extensive research supporting cognitive learning strategies such as retrieval practice, spaced repetition, and elaborative interrogation in improving knowledge retention, these strategies remain underutilized in GME settings [4, 5, 6]. A literature search was conducted to identify the deliberate use and potential efficacy of these learning techniques in EM residencies' curriculum, but none were found. There is a critical educational need to develop and implement novel teaching methodologies that enhance resident learning and retention effectively and efficiently without detracting from patient care. Cognitive learning strategies such as retrieval practice, spaced repetition, and elaboration are potential solutions to this problem and can easily be implemented, complement patient care, and require minimal time investment [6, 7].

## 3 Objective of Innovation

This pilot study aims to address this gap by integrating retrieval practice, spaced repetition, and elaborative interrogation techniques in an innovative, low‐resource curriculum titled “The Breakfast Club.” Prior research in undergraduate and secondary education settings has demonstrated the efficacy of these techniques in promoting deeper understanding and long‐term retention of complex topics [8]. We hypothesize that incorporating these strategies will positively impact EM residents' comprehension and retention of abdominal and gastrointestinal pathophysiology topics.

## 4 Development Process

This pilot study was reviewed and deemed exempt by the HCA Healthcare Institutional Review Board. We conducted a prospective, comparative study to assess the impact of retrieval practice, spaced repetition, and elaboration techniques on knowledge retention and understanding among EM residents (*n* = 21) at a single institution. All levels of training, including first‐year (PGY‐1), second‐year (PGY‐2), and third‐year (PGY‐3) residents, were invited to be participants. Among these residents, those who volunteered to attend *The Breakfast Club* were assigned to the intervention group, and those who did not attend were assigned to the control group.

The intervention group participated in a 1‐h study session that utilized retrieval practice, spaced repetition, and elaborative interrogation techniques, focusing on gastrointestinal pathophysiology. The session engaged learners in active recall and explanatory discussions about selected topics in gastrointestinal pathophysiology. The session content was created by faculty trained in medical education and designed to be resident‐driven and require one faculty facilitator. Residents were responsible for leading the discussion of each question, articulating their reasoning aloud, and identifying areas of uncertainty or disagreement. The faculty facilitator did not provide immediate answers or direct instruction; instead, they guided discussion through targeted prompts, encouraged elaboration on reasoning, and ensured that multiple perspectives were explored. Faculty intervention was limited to clarifying misconceptions that persisted after discussion or highlighting key take‐home points when necessary. The control group did not receive this additional instruction session. Both the intervention and control group participated in the standard residency program curriculum.

To assess knowledge gains, participants completed a 10‐question pretest and postintervention assessment. The pretest, Quiz #1, consisted of 10 MCQ and posttests, Quiz #2 and #3, included five multiple‐choice questions and five short‐answer questions. All questions were developed by expert faculty and reviewed by five additional EM educators to ensure content validity and quality assurance. All quizzes were delivered in an electronic format via an online form that collected participants' e‐mail addresses for identification purposes. There was no set time limit function for the quiz, and it was a closed‐note quiz that was not mandatory for participants to complete.

## 5 The Implementation Phase

### 5.1 Day 0: Baseline Knowledge Assessment

All EM residents at the single institution (*n* = 21) were invited to complete an initial 10‐question multiple‐choice quiz on abdominal and gastrointestinal pathophysiology (Quiz 1). This quiz was utilized to determine the baseline knowledge of the participants. The 10‐question quiz covered five topic areas: infectious processes (Questions 1–2), abdominal wall pathology (Questions 3–4), mechanical processes (Questions 5–6), ischemia (Questions 7–8), and diagnostic testing (Questions 9–10).

### 5.2 Day 1: Intervention Group

On Day 1, the intervention group (*n* = 7) participated in a 1‐h group study session titled *The Breakfast Club*, which took place in the morning, before scheduled didactics in a virtual format. This session utilized retrieval practice, spaced repetition, and elaboration techniques, focusing on topics covered in Quiz #1. The intervention group consisted of four PGY1 residents, three PGY2 residents, and zero PGY3 residents who all voluntarily chose to participate.

The session agenda consisted of a check‐in, single‐word prompt retrieval practice, resident‐driven and faculty‐facilitated elaboration exercises with multiple‐choice questions (MCQs), and a final reflection over the material covered during that session. The session was conducted in a 1‐h time interval.

During the check‐in, the session's expectations and agenda were reviewed to ensure that all residents understood the goals and the application of the retrieval and elaboration techniques. The retrieval practice phase lasted 5 min, during which residents were prompted to either write down something they could recall from their memory only that they had newly learned or reinforced during their last in‐person didactic session, or to choose a topic from a provided board review material list on gastroenterology pathology. They were prompted to retrieve information related to a specific diagnosis or treatment for the selected topic of gastroenterology pathology and then reviewed the retrieved information aloud with the group including the faculty facilitator for accuracy.

The majority of the session (40 min) was dedicated to elaborative interrogation, during which residents worked through and answered five multiple‐choice questions. Each question was constructed in a standard board review format and internally validated by EM residency faculty members. These questions were also on gastrointestinal pathophysiology and covered the same five topics but were unique items from the quizzes. First, the residents individually read the question and answer choices. Then they were prompted to discuss and identify key information in the question stem. Next, the residents as a group reviewed each answer choice, discussing why or why not it was a valid answer choice. Additionally, for any answer choice the residents dismissed, they were asked to elaborate on what potential changes to the question stem could make that choice correct in a future board question. The discussion was resident driven and overseen and guided by the faculty facilitator educated in cognitive learning strategies.

### 5.3 Afternoon: Short Term Retention Assessment—Quiz #2

Later that same day in the afternoon, after receiving didactics on various nongastroenterology pathology educational topics, both the intervention and control groups completed a second quiz (Quiz #2), which included multiple‐choice and short‐answer questions. The second quiz covered the same medical knowledge topics as Quiz #1 but consisted of unique question items in comparison and included an MCQ and short answer prompt on each of the 5 topics.

### 5.4 Day 57: Long Term Retention—Quiz #3

Quiz 3 was provided to all residents 8 weeks after *The Breakfast Club* session and utilized additional unique questions covering the same topics as Quiz #1 and Quiz #2. The outline was the same as Quiz #2, reflecting again the same five topics in gastrointestinal pathophysiology in both MCQ and short answer format.

There were no additional Breakfast Club events and no formal review session discussing the answers to Quiz #1 or Quiz #2 provided between Quiz #2 and Quiz #3. Furthermore, to ensure objective grading, all short‐answer responses were blinded and graded by the same three independent reviewers, with scores averaged according to a predetermined rubric. As 0 PGY3s participated in the intervention, their scores were therefore excluded from the control group quiz calculations. All quiz questions were internally validated by board‐certified EM physicians.

## 6 Outcomes

Overall, both the intervention and control groups demonstrated a trend in knowledge improvement across sequential quizzes. Quiz 1, 2, and 3 had an overall response rate of 95% (20/21). As 0/7 PGY3s participated in the intervention, their responses and scores were excluded from the control group. There was an increase in average multiple‐choice quiz scores in both the intervention group (*n* = 7) and the control group (*n* = 6) between Quiz #1 and Quiz #2 (Table 1). The intervention group, which participated in *The Breakfast Club*, demonstrated the greatest improvement, with mean scores rising from 53% on Quiz #1 to 77% on Quiz #2. This performance also surpassed that of the control group. Although the intervention group's scores declined from 77% on Quiz #2 to 69% on Quiz #3, they remained higher than baseline (Quiz #1) and consistently exceeded the control group's performance.

**TABLE 1:** Average percentage score results on the multiple‐choice questions section for Quizzes #1, #2, and #3 for the intervention and control group. The highest score for each quiz is bolded.

Multiple choice only	Intervention group (*n* = 7)	Control Group (*n* = 6)
Quiz 1 (Baseline)	53%	**55%**
Quiz 2 (Day 1)	**77%**	70%
Quiz 3 (Day 57)	**69%**	63%

Similar to the analysis of the MCQs, both the intervention group and the control group had an increase in their overall percentage score on their short‐term (Quiz #1–Quiz #2) and long‐term follow‐up assessment (Quiz #1–Quiz #3) that included both MCQ and short answer response scores, as shown in Table 2. Additionally, the intervention group had a higher overall score in comparison to the control group for Quiz #2 and Quiz #3. The same pattern is seen in Table 3, which shows the average percentage score for the short answer graded responses for Quizzes #1, #2, and #3 for the intervention and control groups.

**TABLE 2:** Average percentage score results on the overall assessment including multiple choice questions and short answer graded responses for Quizzes #1, #2, and #3 for the intervention and control group. The highest score for each quiz is bolded.

Multiple choice and short answer	Intervention group (*n* = 7)	Control group (*n* = 6)
Quiz 1 (Baseline)	53%	**55%**
Quiz 2 (Day 1)	**64%**	57%
Quiz 3 (Day 57)	**58%**	51%

**TABLE 3:** Average percentage score for the short answer graded responses for Quizzes #1, #2, and #3 for the intervention and control group. The highest score for each quiz is bolded.

Short answer only	Intervention group (*n* = 7)	Control group (*n* = 6)
Quiz 1 (Baseline)	N/A	N/A
Quiz 2 (Day 1)	**51%**	43%
Quiz 3 (Day 57)	**46%**	40%

## 7 Reflective Discussion

Cognitive learning strategies, such as retrieval practice, spaced repetition, and elaboration, are effective and easily implementable strategies to enhance GME. Implementation of *The Breakfast Club* program deliberately exposed participants to these techniques. Improvements in quiz scores both short‐term (Day 1) and long‐term (Day 57) compared to baseline performance were observed for both groups, with the intervention group scoring on average higher on Quiz 2 and Quiz 3 than the control group on both MCQ and short answer responses. Short‐answer responses and scores are presented separately to account for the possibility of correct guessing on multiple‐choice questions, which is less likely with short‐answer prompts. Notably, the intervention group achieved higher scores than the control group on both multiple‐choice and short‐answer questions. These techniques aimed to transform basic, first‐order multiple‐choice questions into more complex, higher‐order assessments through elaboration. Participation in the session was associated with opportunities for learners to identify knowledge gaps in gastrointestinal pathology. While the format shared features with other active learning approaches, the session was intentionally structured around resident‐driven retrieval followed by guided elaborative interrogation, rather than unstructured question review or faculty‐only explanation.

This pilot study has several limitations, including a small sample size at a single institution, a multicomponent intervention, and limited educational content tested. Additionally, the intervention group consisted of voluntary, self‐selected participants, introducing potential selection bias, as these residents may have been more internally motivated or more interested in medical education.

It is also worth noting that the spaced quizzes themselves served as a form of spaced repetition for both intervention and control groups, as they tested the same content at different intervals. Therefore, the spaced repetition technique alone cannot be isolated as a *Breakfast Club* only cognitive learning strategy intervention in this study. The retrieval practice and elaborative interrogation portions however were unique to the intervention group. Although the session was conducted virtually, it could easily be adapted for in‐person delivery and requires minimal resources (e.g., PowerPoint, paper, and writing utensils). The *Breakfast Club* session lasted 1 h, making it feasible to integrate into an existing didactic schedule. If desired, the session could be extended to cover additional topics within gastrointestinal pathophysiology or adapted to other areas of GME.

Future studies should ideally be multi‐institutional to increase sample size and statistical power, with randomization into intervention and control groups to reduce selection bias. Given the small sample size in this study, it remains unclear whether the observed trends would achieve statistical significance in a larger cohort. Additional research is needed to evaluate whether these cognitive learning strategies can consistently improve learning outcomes in GME settings and if these strategies can have synergistic effects.

## Author Contributions

**Abigail Alorda:** conceptualization, investigation, writing – original draft, methodology, writing – review and editing, project administration, data curation, resources. **Joseph R. Ray:** validation, data curation. **Stephanie Cohen:** validation, data curation. **Shayne Gue:** investigation, writing – review and editing, data curation, supervision, formal analysis, validation.

## Funding

The authors have nothing to report.

## Ethics Statement

This study was conducted in accordance with the Declaration of Helsinki and exempt by the HCA Healthcare Centralized Algorithms for Research Rules on IRB Exemptions (CARRIE).

## Consent

The authors have nothing to report.

## Conflicts of Interest

The authors declare no conflicts of interest.

## Data Availability

The datasets used and/or analyzed during the current study are available from the corresponding author on reasonable request.
